# Are full-face helmets the most effective in preventing head and neck injury in motorcycle accidents? A *meta*-analysis

**DOI:** 10.1016/j.pmedr.2020.101118

**Published:** 2020-05-13

**Authors:** Soramon Chaichan, Thatchanon Asawalertsaeng, Pat Veerapongtongchai, Paiboon Chattakul, Sittichai Khamsai, Patnarin Pongkulkiat, Verajit Chotmongkol, Panita Limpawattana, Jarin Chindaprasirt, Vichai Senthong, Chetta Ngamjarus, Yuwares Sittichanbuncha, Amnat Kitkhuandee, Kittisak Sawanyawisuth

**Affiliations:** a Faculty of Medicine, Khon Kaen University, Khon Kaen, Thailand; b Department of Medicine, Faculty of Medicine, Khon Kaen University, Khon Kaen, Thailand; c Department of Epidemiology and Biostatistics, Faculty of Public Health, Khon Kaen University, Khon Kaen, Thailand; d Department of Emergency Medicine, Faculty of Medicine Ramathibodi Hospital, Mahidol University, Bangkok, Thailand; e Department of Surgery, Faculty of Medicine, Khon Kaen University, Khon Kaen, Thailand

**Keywords:** Prevention, Road traffic accidents, Risk factors

## Abstract

Motorcycles are the most common type of vehicle involved in traffic deaths in developing countries. Although helmets can provide protection against injury, there is limited evidence available regarding which type of helmet best protects against head and neck injuries in this setting. This review was conducted based on articles in the PubMed, Scopus, and Web of Science databases. We compared full-face helmets with other types of helmet with regard to head and neck injury prevention in road accidents involving motorcyclists. Of 702 studies, six were eligible with a total of 6,529 participants. When compared with partial and open helmets, the odds ratio of full-face helmets was 0.356 (95% CI of 0.280, 0.453) and 0.636 (95% CI of 0.453, 0.894), respectively, for reduction of head and neck injuries. In conclusion, full-face helmets reduced head and neck injuries in motorcycle accidents to a greater extent than other types of helmet. Policy makers should recommend that motorcyclists use full-face helmets.

## Highlights

• Full-face helmet has lower head/cervical injury than half-coverage helmet.
• Full-face helmet has lower head/cervical injury than open helmet.
• Full-face helmet has lower head/cervical injury than other types of helmet.
• Motorcyclists may consider wearing full-face helmet.

## 1 Introduction

Motorcycles are the most common type of vehicle involved in traffic deaths in developing countries (Erenler and Gümüş, 2019). The Institute for Health Metrics and Evaluation reported that in 2016, road injuries were the leading cause of death and disability and were ranked as the second most common cause of premature death in Thailand (Institute for Health Metrics and Evaluation, 2018). The WHO reported that road traffic deaths were highest in Africa and South-East Asia in 2016, with rates of 26.6 and 20.7/100,000 people, respectively (Global status report on road safety, 2018).

Studies spanning previous decades have found head injury to be the most common type of injury in autopsied victims of motorcycle accidents (41.4%) (Faduyile et al., 2017). A 2017 report from Nigeria also found that craniocerebral injuries were the cause of death in 50.7% of motorcycle fatalities (Faduyile et al., 2017). A Cochrane review found that wearing a helmet protected against death and head injury with significant odds ratios of 0.58 and 0.31, respectively (Liu et al., 2008).

There are three common types of helmet: full face, open face, and half (or partial) coverage. The motorcycle helmet laws in many countries do not specify helmet type. A study from Korea found that only full face and open face helmets significantly reduced head injuries in motorcycle accidents with a coefficient of −0.368 (p < 0.001) and −0.235 (p 0.040), respectively (Sung et al., 2016). However, half-coverage helmets did not significantly lower the risk of head injury (p value 0.101). A Cochrane review published in 2008 found that there were insufficient data to conclude which helmet type was most effective in reducing the risk of injury. This is because the five studies included in the *meta*-analysis did not show significant differences in terms of head or cervical injuries between full-face and open-faced helmets, with odds ratios ranging between 0.76 and 1.13 (Liu et al., 2008). This study, thus, aimed to determine the most effective helmet type in preventing head and cervical injuries in motorcycle accidents. These data may be useful in shaping future helmet laws.

## 2 Methods

A literature review was followed by a systematic search of Cochrane reviews published on PubMed, Scopus, and Web of Science databases (March 3, 2020). The eligible studies were those 1) that compared full-face helmets with other types of helmets in motorcycle accidents, 2) in which the outcomes involved head or cervical injuries, and 3) were published in English. The study types included randomized controlled trials, controlled trials, cohort/retrospective cohort studies, case-control studies, and descriptive studies (either prospective or retrospective). Those studies with ecological designs, case series, or for which the full text was unavailable were excluded. There were no limits with regard the age or sex of the participants in the eligible studies. The search keywords that were used were as follows: motorcycle, accident(s), helmet, head injury/injuries, and cervical injury/injuries (supplemental file). Eligible studies were those that compared full-face helmets with other types of helmet.

The studied variable was helmet type, and the outcome variables included any head or cervical injury including traumatic brain injury, brain contusion, facial fracture, and cervical spine injury. For studies with several outcomes, only the outcomes mentioned above were selected for inclusion in the analysis (determined based on severity and frequency). The numbers of patients with head or cervical injuries were the primary end point and were tabulated by type of helmet. The full-face helmet was used as the primary type and was compared with other types of helmet based on the primary outcome. We summarized all eligible studies. The odds ratio and 95% confidence interval (CI) of full-face helmets were calculated based on comparisons with other types of helmet. The odds ratios were calculated using the traditional method and Review Manager (RevMan) Software version 5.3 with a fixed method. Forest plots for each comparison and I^2^ are also shown.

## 3 Results

**Fig. 1:**
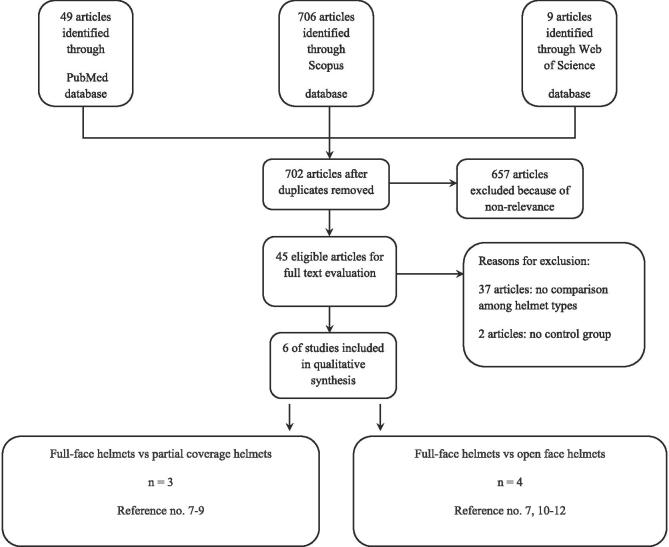
Flow chart of article search using keywords to evaluate helmet types on the prevention of head and neck injuries in motorcycle riders.

Searches of the three databases resulted in 764 articles (see appendix 1 for a list of the search terms used), 702 of which remained after duplicate removal. Of these, 657 were excluded due to non-relevance, leaving 45 eligible articles for full text evaluation. Thirty-nine of these were excluded for the reasons shown in Fig. 1. The remaining 6 articles were included in the analysis with a total of 6,529 participants. These articles were categorized as either full-face versus half-coverage helmet comparison (n = 3) (Lam et al., 2015, Ramli et al., 2014, Yu et al., 2011) or full-face versus open-face helmet comparison (n = 4) (Yu et al., 2011, Cini et al., 2014, Hitosugi et al., 2004, Lopes Albuquerque et al., 2014). Note that one study included both comparisons (Yu et al., 2011). The characteristics of the included studies are summarized in Table 1, Table 2 and listed according to helmet comparison.

**Table 1:** Summary of studies comparing full-face and half-coverage helmets with regard to head and cervical outcomes in motorcyclists who had road accidents.

Factors/Study	Lam et al	Ramli et al	Yu et al
Country	Taiwan	Malaysia	Taiwan
Year	2015	2014	2011
Study design	Case-control	Case-control	Matched case-control
Inclusion	- patients with ICD-9 codes 800–804, 850–854 (brain concussion, intracranial hemorrhage, skull-bone fracture) - motorcycle crash - over 17 years of age	- all motorcyclists or passengers - all ethnic groups - all age groups and genders - all injury types and levels of severity - were involved in a motorcycle crash in the Catchment area (southern Klang Valley) during the study period (2010–2011)	- Age > 15 y - Lived in Taichung - Visited the emergency room at China Medical University Hospital due to motorcycle injuries
Exclusion	Any cases with missing data on helmet use, helmet type, or cervical spine injury	Motorcyclists who did not sustain any injury, or discharged themselves from hospital care without a definitive diagnosis, and those involved in road crashes outside Klang Valley	Riders who were not operating a motorcycle—i.e. those who were riding a minibike, a bicycle or a tricycle or wore a safety helmet for construction or were involved in a crash outside the city of Taichung
Numbers of participants	5,225 patients; 173 (3.3%) case group and 5,052 (96.7%) control group	755 participants; 391 (51.8%) facial injuries and 364 (49.2%) no facial injury	458 pairs of case-control; not all helmeted
Primary outcome	Cervical spine injury	Facial injury	Head injury
Full-face helmet with head injury, n	28	6	50
Full-face helmet without head injury, n	1,259	12	73
Half-coverage helmet with head injury, n	104	304	274
Half-coverage helmet without head injury, n	3,385	293	208

**Table 2:** Summary of studies comparing full-face and open helmets on head and cervical outcomes in motorcyclists who had road accidents.

Factors/Study	Hitosugi et al	Cini et al	Lopes Albuquerque et al	Yu et al
Country	Japan	Brazil	Brazil	Taiwan
Year	2004	2014	2014	2011
Study design	Retrospecitve study	Case-control	Retrespective cohort	Matched case-control
Inclusion	Jikei University autopsies of motorcyclists who died in traffic accidents from 1998 to 2002	Patients with facial injuries from a motorcycle accident	Motorcycle accident victims who had to be referred to the outpatient clinic at the hospital	As in Table 1
Exclusion	NA	Those with injuries to any other part of the body or whose injuries resulted in death	Incomplete hospital records or refusal to participate	As in Table 1
Numbers of participants	36	1,628	253	458 pairs
Primary outcome(s)	Number of severely injured body regions	Facial injuries	Facial Injury Severity Scale, traumatic brain injury, facial fractures	Head injury
Full-face helmet with head or cervical injury, n	9*	12**	24***	50
Full-face helmet without head or cervical injury, n	8*	63**	22***	73
Open helmet with head or cervical injury, n	16*	9**	39***	106
Open helmet without head or cervical injury, n	3*	25**	12***	149

Note. NA: not available; * indicates severe head injury; ** indicates zygomatic fracture; ***indicates traumatic brain injury.

**Fig. 2:**
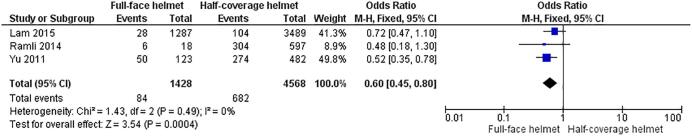
Comparison of full-face helmets and half-coverage helmets with regard to head and cervical outcomes in motorcyclists who had road accidents.

*Full-face* versus *half-coverage helmet comparison*. There were two studies from Taiwan and one study from Malaysia that compared full-face and half-coverage helmets. The outcomes were facial injury, traumatic brain injury, and cervical spine injury (Table 1). There were a total of 5,996 participants in all of the half-coverage helmet studies, 766 of whom experienced one or more of these outcomes, and 5,230 of whom did not (Fig. 2). The odds ratio of full-face over half-coverage helmet was 0.356 (95% CI of 0.280, 0.453) with a p value of < 0.001. The odds ratio computed using RevMan was 0.60 (95% CI of 0.45, 0.80; p value < 0.001 with I^2^ of 0%; Fig. 2).

**Fig. 3:**
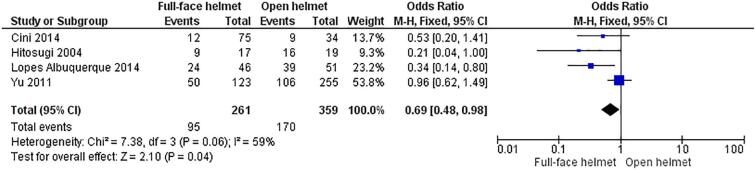
Comparison of full-face helmets and open helmets with regard to head and cervical outcomes in motorcyclists who had road accidents.

*Full-face* versus *open helmet comparison*. There were four studies in the analysis that compared full-face and open helmets: two from Brazil, one from Japan, and one from Taiwan. Three of these studies had traumatic brain injury or severe head injury as an outcome. The other study (from Brazil) had several outcomes including facial contusion, zygomatic fracture, nasal fracture, mandibular fracture, orbital fracture, dentoalveolar fracture, and jaw fracture (Cini et al., 2014). Zygomatic fracture was selected to be included in the analysis due to it being both more severe and more common than the other outcomes (Table 2). There were a total of 620 participants in these four studies, 265 of whom had experienced one or more of the outcomes mentioned above, and 355 of whom had not (Fig. 3). The odds ratio of full-face helmets was 0.636 (95% CI of 0.453, 0.894) with a p value of 0.006. The odds ratio computed using RevMan was 0.69 (95% CI of 0.48, 0.98; p value 0.04 with I^2^ of 59%; Fig. 3).

**Fig. 4:**
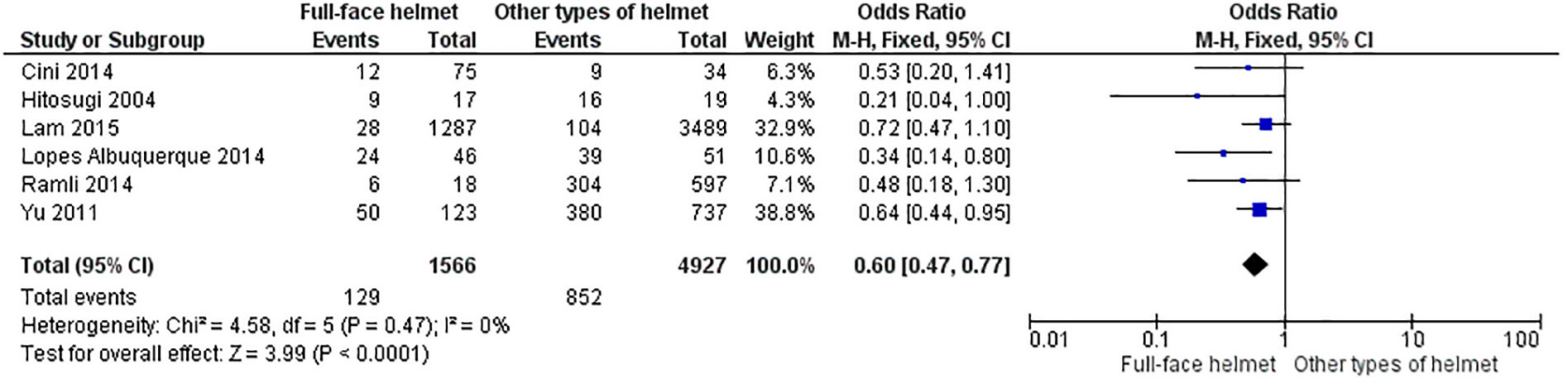
Comparison of full-face helmets and other types of helmet with regard to head and cervical outcomes in motorcyclists who had road accidents.

*Full-face helmets* versus *other types of helmet*. The total number of participants in all six studies was 6,529. This is excluding duplicate participants in a Taiwanese study by Yu (n = 123), all of whom had worn full-face helmets (head injury [n = 50], no head injury [n = 73]), as shown in Fig. 4. Full-face helmets had an odds ratio of 0.429 (95% CI of 0.352, 0.524) with a p value of < 0.001. The odds ratio computed using RevMan was 0.60 (95% CI of 0.47, 0.77; p value < 0.001 with I^2^ of 0%; Fig. 4).

## 4 Discussion

This review had a large sample size (6,529 participants) and found that full-face helmets were the most effective at preventing head and cervical injuries in motorcycle accidents (Fig. 4). Full-face helmets had significant protective effects on the outcomes compared with either half-coverage helmets (Fig. 2) or open helmets (Fig. 3).

Each helmet type has its own advantages and disadvantages. The full-face helmet has no articulation, but it may be heavier or cause discomfort and limitations with regard to visibility. Half-coverage or open helmets tend to be lighter but require articulation during use. Therefore, a rider's choice of helmet type may depend on individual preference or local regulations (Dapilah et al., 2017). Two studies – one from Brazil and one from Iran – reported that more motorcyclists wore full-face helmets than open helmets (69% in Brazil and 76% in Iran) (Cini et al., 2014, Amirjamshidi et al., 2011). However, the rate of full-face helmet use was only 2.4% in a study from Malaysia (Ramli et al., 2014). A study from Australia found that full-face helmets may result in a somewhat lower rate of cervical spine injury than open helmets (14.4% vs 18.2%) (O'Connor et al., 2002).

The main finding of this review is that full-face helmets were better than other types of helmet at preventing head and cervical injuries in motorcycle accidents. All analyses were compatible between traditional and RevMan calculations. The risk of head and cervical injuries for riders who used full-face helmets was 64% lower compared with those who used half-coverage helmets (Fig. 2), 36% lower than in those who used open helmets, and 57% lower when compared with both those who used half-coverage helmets and those who used open helmets (Fig. 4). A study from Malaysia showed that factors were significantly associated with facial injuries in motorcycle accidents: helmet use and helmet fixation (Ramli et al., 2014), of which helmet fixation had the greatest effect. Full-face helmets provided greater fixation than the other articulated helmets. Additionally, riders in Thailand are five times more likely to remove their helmet prior to a traffic accident than those in the US (25% vs 5%) (Ouellet and Kasantikul, 2006).Wearing an open or half-coverage helmet may make it easier to remove. However, full-face helmets may cause discomfort due to the greater heat and humidity in tropical countries like those in Southeast Asia (de Rome et al., 2012). Nevertheless, if a rider wears a full-face helmet, his/her risk of head and neck injury will likely be lower than if he/she uses an open/half-coverage helmet.

There were some limitations in this study. First, the six studies included in the analysis were from only four countries: two from Taiwan, two from Brazil, one from Malaysia, and one from Japan. Second, the definitions of head and cervical injury were not uniform among the studies, particularly in those that compared full-face and open helmets (Fig. 3). In addition, the eligibility criteria for participants varied among the studies. Most of the studies enrolled patients involved in motorcycle accidents, but the study by Lam et al. enrolled all ICD-9 patients (n = 5,225) (Lam et al., 2015), and another enrolled autopsied cases (n = 36) (Hitosugi et al., 2004). Third, comparisons of full-face versus open helmet had high heterogeneity as calculated using RevMan (I^2^ of 59%). Finally, the outcomes focused only on head and cervical injury and did not include other parts of body. However, these types of injuries accounted for over 50% of injuries motorcycle accident victims. The analysis in this study was also not adjusted for other factors such as severity of crash.

## 5 Conclusions

Full-face helmets reduced head and neck injuries in motorcycle accidents to a greater extent than other types of helmet. Policy makers should recommend that motorcyclists use full-face helmets.

## Financial disclosure

The authors have nothing to disclose.

## CRediT authorship contribution statement

**Soramon Chaichan:** Conceptualization, Methodology, Formal analysis, Investigation, Data curation, Writing - original draft. **Thatchanon Asawalertsaeng:** Methodology, Formal analysis, Investigation, Visualization. **Pat Veerapongtongchai:** Methodology, Formal analysis, Investigation, Visualization. **Paiboon Chattakul:** Methodology, Investigation, Visualization, Supervision. **Sittichai Khamsai:** Methodology, Investigation, Visualization, Supervision. **Patnarin Pongkulkiat:** Methodology, Investigation, Visualization, Supervision. **Verajit Chotmongkol:** Methodology, Investigation, Visualization, Supervision. **Panita Limpawattana:** Methodology, Investigation, Visualization, Supervision. **Jarin Chindaprasirt:** Methodology, Investigation, Visualization, Supervision. **Vichai Senthong:** Methodology, Investigation, Visualization, Supervision. **Chetta Ngamjarus:** Methodology, Investigation, Visualization, Supervision. **Yuwares Sittichanbuncha:** Methodology, Investigation, Visualization, Supervision. **Amnat Kitkhuandee:** Conceptualization, Methodology, Investigation, Visualization, Supervision, Project administration, Writing - review & editing. **Kittisak Sawanyawisuth:** Conceptualization, Methodology, Investigation, Visualization, Supervision, Project administration, Writing - review & editing.

## Declaration of Competing Interest

The authors declare that they have no known competing financial interests or personal relationships that could have appeared to influence the work reported in this paper.
