# Supplementary material for: Are full-face helmets the most effective in preventing head and neck injury in motorcycle accidents? A meta-analysis
Source: Prev Med Rep. 2020 May 13;19:101118. doi: 10.1016/j.pmedr.2020.101118 (PMC7264075; doi:10.1016/j.pmedr.2020.101118)
Supplement: Supplementary data 2 [file mmc2.docx]

**Appendix**

**Searching result in PubMed on 3 March 2020 (found 49 articles)**

1. " head protective devices"[MeSH Terms] OR helmet[Text Word]
2. "motorcycles"[MeSH Terms] OR motorcycle[Text Word]
3. accident*[Title/Abstract]
4. (#2 AND #3)
5. head injury[Title/Abstract]
6. (#1 AND (#4 AND #5))

**Searching result in Scopus on 3 March 2020 (found 706 articles)**

1. ( TITLE-ABS-KEY ( "head protective devices"  OR  helmet ) )  ( ( ( TITLE-ABS-KEY ( motorcycle* ) )  AND  ( TITLE-ABS-KEY ( accident* ) ) )  AND  ( TITLE-ABS-KEY ( head  AND injury ) ) )

**Searching result in Web of Science on 3 March 2020 (found 9 articles)**

1. ALL FIELDS: (head protective devices)
2. ALL FIELDS: (helmet)
3. #2 AND #1
4. TS=(motorcycle*)
5. TS=(accident*)
6. #5 AND #4
7. TOPIC: (head injury)
8. #7 AND #6 AND #3
